# Influence of Adding Modifying Elements and Homogenization Annealing on Laser Melting Process of the Modified AlZnMgCu with 4%Si Alloys

**DOI:** 10.3390/ma14206154

**Published:** 2021-10-17

**Authors:** Ahmed O. Mosleh, Asmaa M. Khalil, Irina S. Loginova, Alexey N. Solonin

**Affiliations:** 1 Department of Physical Metallurgy of Non-Ferrous Metals, National University of Science and Technology “MISiS”, Leninsky Prospekt 4, 119049 Moscow, Russia; asmaa.mostafa@feng.bu.edu.eg (A.M.K.); loginova@misis.ru (I.S.L.); solonin@misis.ru (A.N.S.); 2 Department of Mechanical Engineering, Shoubra Faculty of Engineering, Benha University, Shoubra St. 108, Cairo 11629, Egypt; 3 Department of Heat Treatment and Physics of Metals, Ural Federal University, Mira St. 19, 620002 Ekaterinburg, Russia

**Keywords:** AlZnMgCu, laser melting, modifying elements, solidification cracks, microhardness

## Abstract

AlZnMgCu, the high-strength aluminum alloy, is unsuitable for laser melting applications due to its high hot cracking sensitivity and large solidification temperature range. Adapting this alloy for laser melting processing is a high-demand research issue for extending its use. Thus, this paper investigates the effect of adding 4%Si, 4%Si-Sc+Zr, 4%Si-Ti+B, and homogenization annealing on the laser melting process (LMP) of AlZnMgCu alloy. Homogenization annealing at 500 °C for 6.5 h was selected to dissolve most of the low melting temperature phases into the grain matrix and perform stable alloys for the LMP. The pulsed laser melting process (PLM) was performed on the as-casted and the homogenized samples. The microstructures of the as-casted, the homogenized alloys, and after the LMP were evaluated. In addition, the hardness of the base metal (BM) and laser melted zone (LMZ) were measured. The results revealed that the microstructure was enhanced and refined in the as-cast state by adding the modifiers due to the increasing nucleation potency of solidification sites and the formation of primary Al_3_(Ti, Zr, Sc) phases. The average grain size was decreased by 15.6 times when adding 4%Si + 0.4%Zr + 0.29%Sc, while it decreased by 10.2 times when adding 4%Si + 1%Ti + 0.2%B. The LMZ of the as-casted samples exhibited a non-uniform distribution of the grains and the elements after the LMP. This was attributed to the evaporation of Zn, Mg during the high laser power process besides the non-uniform distribution of elements and phases in samples during casting. After the laser treating of the homogenized samples with 4%Si-Sc + Zr, uniform columnar grains were formed in the direction of the laser. The presence of Ti and B changed the crystallization nature, resulting in the LMZ with very fine and equiaxed grains due to forming many nucleation centers during solidification. The hardness values have positively increased due to Si addition and adding a combination of Ti + B and Sc + Zr. The maximum hardness was 153.9 ± 5 HV achieved in the LMZ of the homogenized samples of 4%Si + 1%Ti + 0.2%B.

## 1. Introduction

Al 7XXX alloys are extensively used to manufacture structural components in various industries, including the aerospace industries, because of their excellent stiffness-to-weight ratios, good machinability, and strength-to-weight. The geometric complexity of many aerospace components posed challenges in conventional subtractive manufacturing methods. However, the LMP is extensively utilized in this field because of the small size, high value, and geometric complexity of the manufactured components. Furthermore, LMP techniques decrease the overall amount of aircraft parts by developing and producing complicated topologies [1,2,3].

The chemical composition of the high-strength age-hardenable (7xxx series) aluminum alloys renders them unsuitable for laser melting [4,5]. These alloys include alloying elements that significantly increase the solidification temperature range and crack sensitivity [6,7]. The tensile deformation can be induced in the inter-dendritic semi-solid due to the solidification shrinkage and the thermal contraction, resulting in solidification cracks along the full length of the columnar grains [8,9,10]. LMP has been found to produce extensive cracking in these alloys, and one challenge is knowing the reason for the formation of cracks and how to stop them from forming [11,12,13]. Adding modifiers and alloying elements was considered to be a viable solution to this issue [14,15,16,17,18].

Among 7xxx series alloys, AA7075 is the most common commercial aluminum alloy due to its good machinability and medium strength or high strength. Many attempts have been expended to create a crack-free alloy with high performance; however, hot cracks and pores formation are extensive during laser melting [19,20]. Further, the evaporation of Zn and Mg during the LMP of the AA7075 often results in varied compositions throughout the processed material [21]. To enhance the processability of Al alloys in LMP and control the microstructure and characteristics of the processed material, a variety of approaches, such as grain refining and controlling the solidification rate, have been used [6,7,16]. Martin et al. [4] showed that the crack formation could be resolved by adding nanoparticles of nucleates during the laser additive manufacturing to control the solidification. Cavities and hot cracks were discovered to be caused by the solidification shrinkage of the inter-dendritic liquid retained between dendritic grains [4]. Equiaxed grains, which behave like a low-resistance granular solid, can reduce the influence of the retained liquid. Fine grains maximize the total grain boundary area per unit volume, which hardens the material and eliminates the intergranular cracks [22]. 

The most common grain refinement method in casting is adding Ti, B, Sc, and Zr elements. Adding Ti and B produces TiB_2_ inoculant particles, including an Al_3_Ti layer to facilitate nucleation [23,24]. The inclusion of Zr has also been shown to improve the microstructure of Al alloys. Al_3_Zr particles develop initially in the melt during solidification. The Al_3_Zr particles offer heterogeneous nucleation sites for the main Al phase [14,25,26,27,28,29]. Adding Sc, especially for Al-Mg alloys, has also been used to improve the grain structure and alloy for different applications and technologies [30].

In the same way, as Zr particles, Al_3_Sc particles offer heterogeneous nucleation sites [31]. The hot cracking potential during the LMP can be minimized by 7075 alloy modification by adding Si [32,33]. Silicon is a common and cheap aluminum alloying component that eliminates the microcracks in 7075 alloys processed using the LMP [34]. According to Sistiaga et al. [6], using a powder produced by combining 7075 with 4% Si particles prevents microcracks in additively manufactured products. Aversa et al. [35] produced crack-free selective laser melting samples from powder made by combining 50% 7075 and 50% AlSi10Mg powders. 

AA7075 alloy was selected for this research due to its very good cast and wrought state properties. Still, the hot cracks were the main limitations for this alloy during the high laser process, such as laser welding, melting, and additive manufacturing. Thus, this study aimed to adapt the high-strength aluminum alloy AA7075 for the laser melting processes by obtaining a fine equiaxed and defect-free structure, especially the hot cracks. And to study the effect of the additional elements, heat treatment, and laser melting processing on the structure and properties of the investigated alloys under the rapid solidification conditions.

## 2. Materials and Methods

The chemical compositions of the investigated alloys listed in Table 1 were melted in graphite-fireclay crucible (Aug. Gundlach KG, Grossalmerode, Germany) using a 5.5 kW 7 kg Nabertherm resistance furnace (Nabertherm, Lilienthal, Germany) at a temperature of 825 °C. Pure aluminum grade A95, pure Zinc, pure magnesium, and master alloys of Al-10% Mn, Al-2% Sc, Al-3.5% Zr, Al -10% Cr, Al-5% Ti-1% B, Al-12%Si, and Al-53.5% Cu were used for alloy preparation. The melted composition was stirred to homogenize the alloying elements with the base melt alloy, and then the melted metal was poured into a water-cooled copper mold. The THERMO-CALC software with TCAL4 database (Thermo-Calc Software, Stockholm, Sweden) was utilized to create phase mass fraction-temperature dependence diagrams for each alloy’s nominal composition after the casting process. In an Argon environment, a Setaram Labsys (SETARAM Instrumentation, Caluire, France) calorimeter was used to perform a differential scanning calorimetry (DSC) analysis with a heating rate of 5 °C/min. Temperatures ranging from 20 °C to 1000 °C were used in the experiments.

Before laser melting processing, the casted samples were homogeneously annealed at 500 °C for 6.5 h for modified alloys with 4%Si to ensure full dissolving of the intermetallic phases. For LMP, the samples were cut from the ingots with a thickness of 1.5–2 mm, were cleaned and polished using SiC papers with numbers P800-P1200 on Struers LaboPol-5 machine (Struers APS, Ballerup, Denmark), then were treated with a 10% aqueous solution of NaOH and a 15% aqueous solution of HNO_3_. After removing the oxide film and brightening the surface, rinsing was performed in running water and drying to remove moisture. A MUL-1-M-200 pulse-periodic laser welding machine (OOO Latikom, Moscow, Russia) with an Nd: YAG laser operating with a wavelength of 1064 nm under protective Argon gas was used laser melting process. The processing parameters for tracks were tabulated in Table 2. These parameters were used to melt the alloys with a single track.

The laser-treated samples were polished in two positions for microstructure examinations: the first one, parallel to the surface melting tack. In the second position, the samples were cut and polished perpendicular to the laser melting tracks. The samples were polished using SiC papers with numbers ranging from P320 to P4000 using a Struers machine “Labopol-5” (Struers APS, Ballerup, Denmark). The polished surface was electrically etched at 18V using a 10% electrolyte (saturated solution of H_3_BO_3_ in HF) in distilled water. Optical microscope (OM), Axiovert 200 MMAT, (Carl Zeiss, Oberkochen, Germany) and scanning electron microscope, Tescan-VEGA3, (Tescan Brno s.r.o., Kohoutovice, Czech Republic) were used to examine the microstructure of the as-cast and after the LMP. The linear intercept technique was used to calculate the average grain size. A Vickers hardness testing machine measured microhardness values of the LMZ with a load of 25 gf and a shutter speed of 15 s. Hardness results were obtained using an HVD-1000AP hardness tester machine (Wolpert Wilson Instruments, Aachen, Germany) with HV5, a load of 500 g, and a shutter speed of 10 s.

## 3. Results and Discussion

### 3.1. Characterization of the Modified Alloys

#### 3.1.1. Thermo-Calc Prediction

**Figure 1:**
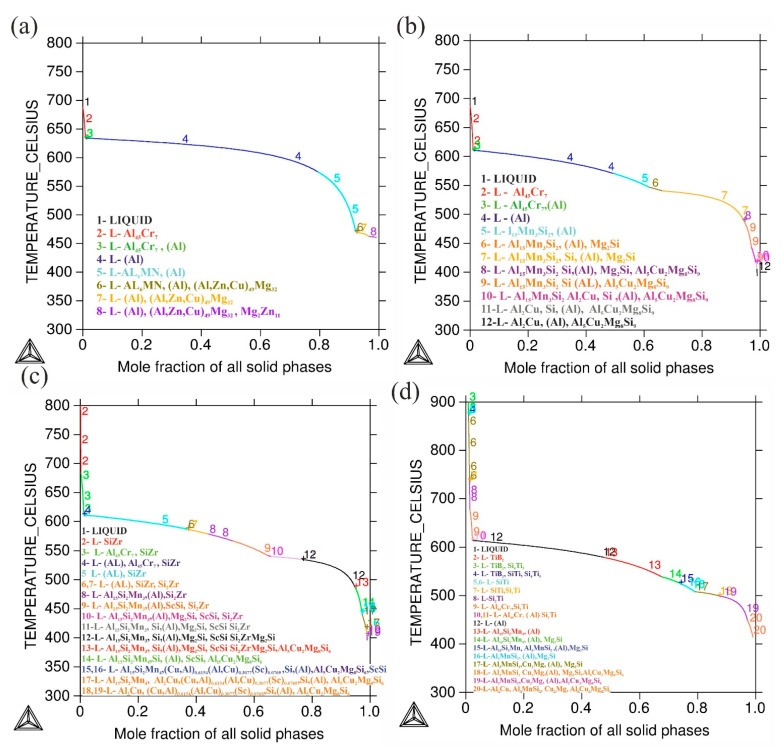
Thermo-Calc generated Scheil solidification curve with predicted phases for (**a**) 7075 alloy, (**b**) 7075-4%Si, (**c**) 7075-4%Si + ScZr, and (**d**) 7075-4%Si + TiB.

As stated above in the introduction, one of the main issues for LMP of the 7075 alloys is their high tendency of hot cracking, which is related to solidification shrinkage. Si is added initially to provide an additional liquid phase for reducing the tendency of solidification cracking and decreasing solidification shrinkage as shown in Si-rich Al alloys like Al-Si and AlSi10Mg alloys, which may be successfully additively manufactured [6,34,35]. Figure 1 illustrates the solidification curves with the predicted phases produced by the Thermo-Calc database of the investigated alloys. Figure 1a–d shows that adding 4%wt Si to the AlZnMgCu alloy would reduce the dfs/dt value (=slope of the solidification curve), where t is the solidification time, and fs is a solid phase fraction. Generally, according to the computed solidification curves, the inclusion of 4%wt Si reduces the temperature difference between solidus and liquidus temperatures. According to Kou et al. [36], a higher temperature difference between the liquidus and the solidus temperatures increases cracks. That is, the presence of Si decreased the susceptibility to cracks by altering the solidification behavior of the basic AlZnMgCu alloy, according to the researchers. Adding Ti + B, and Zr + Sc, insignificant affect the solidification curves (Figure 1c,d). The solidification range of the alloys after adding Sc + Zr and Ti + B to 7075-4%Si alloy was decreased by 8 °C and 12 °C respectively.

#### 3.1.2. Microstructure Evaluation in As-Cast Condition

**Figure 2:**
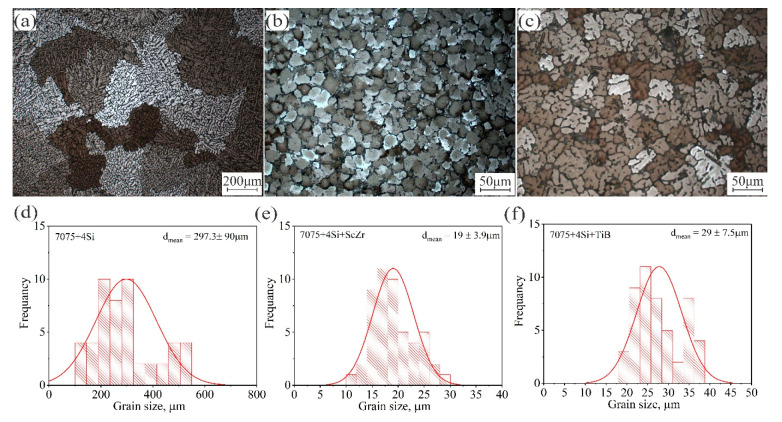
Optical micrographs and grains statistical of the as-casted alloys; (**a**,**d**) 7075-4%Si, (**b**,**e**) 7075-4%Si + ScZr, and (**c**,**f**) 7075-4%Si + TiB.

Figure 2 presents the microstructure and grains statistical of the as-cast modified alloys. The microstructure of the 7075-4%Si has a formation of primary dendrites of the Al-rich solid solution with a coarse-grained dendritic structure (Figure 2a) with an average grain size of 297 ± 90 µm. Adding 0.29%Sc and 0.4%Zr, the structure changed to a very fine nondendritic structure according to the effect of the primary Al_3_(Sc, Zr) phase as a refining source and the suppression of grain growth, shown in Figure 2b, with a grain size of 19 ± 3.9 µm. Adding 1% Ti–0.2% B resulted in uniform and fine dendritic grains due to the soluble Al_3_Ti and TiB_2_, which act as potential nuclei for primary phase nucleation. The average grain size was 29.2 ± 7.5 µm shown in Figure 2c. Figure 2d–f shows the grains size frequency of the investigated alloys. It was observed that the normal distribution of grains was narrower in the case of 7075-4%Si + ScZr (Figure 2e) than in those of the other alloys. The narrow normal distribution of grains confirms the equiaxed uniform grains. The as-cast 7075-4%Si alloy exhibited non-uniform equiaxed grains resulted in the wide normal distribution of grains. The grain size of 7075-4%Si is 297 ± 90 µm and 19 ± 3.9 µm in 7075-4%Si + ScZr, and from 29 ± 7.5 µm in 7075-4%Si + TiB. Grains morphology, size, and frequency play a significant role in the performance of the material under the different processes. 

**Figure 3:**
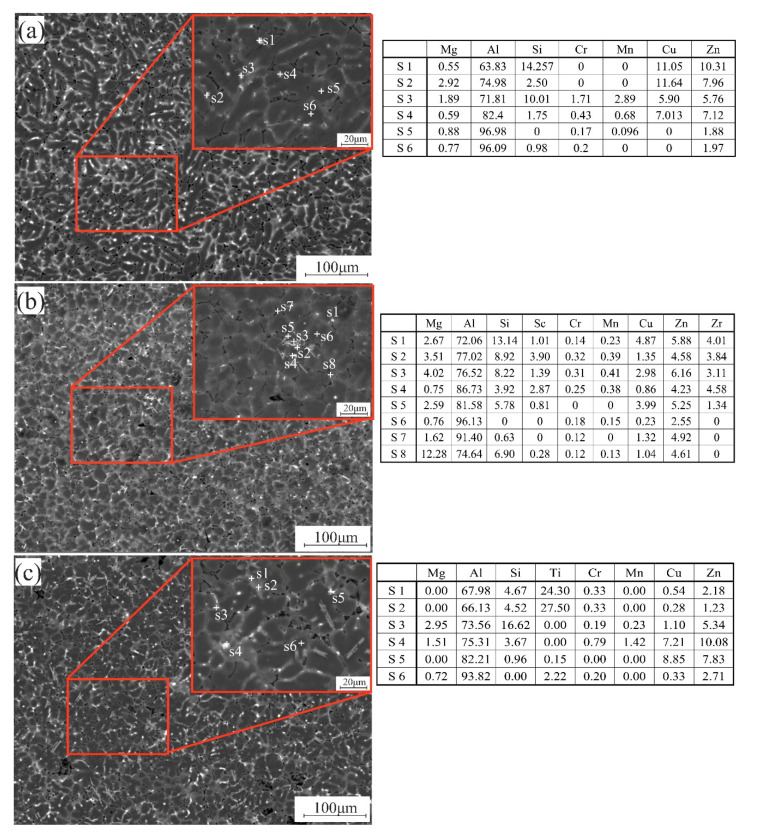
SEM micrographs and EDS analysis for the formed phases of the as-casted samples; (**a**) 7075-4%Si, (**b**) 7075-4%Si + ScZr, and (**c**) 7075-4%Si + TiB.

According to SEM and EDS analysis in Figure 3, the bright zones are the non-equilibrium phases, and the dark zones are the primary solid solution. 7075-4%Si has a dendrite cell of Al solid solution with small amounts of Cu, Mg, Si, Cr, Mn, and Zn, as observed in Figure 3a. However, it can be observed that the intermetallic phases were formed in the inter-dendritic regions. The formed phases besides the Al solid solution and primary Si were Al_2_Cu, MgZn_2_, and Mg_2_Zn_11_. Adding Sc + Zr to 7075-4%Si can form the primary aluminide Al_3_(Sc, Zr) particles and the eutectic phase of Si, Mg, Cu, Mn, and Zn in the inter-dendritic areas (Figure 3b). The addition of Ti and B led to a uniform distribution of the liquid phase with the insoluble TiB_2_ particles and the soluble Al_3_Ti, which modified the grain structure (Figure 3c). These formed phases were confirmed by Thermocalc data. The presence of Al_3_(Sc, Zr, Ti) enhanced the nature of the crystallization of the alloy. These nucleant particles lead to an equiaxed microstructure formation with a uniform distribution of small grains and porosity, leading to improved mechanical and fatigue properties [37]. During casting, grain refiners were added to the melt to reveal TiB_2_ and Al_3_Ti particles, which act as heterogeneous nuclei for the primary Al. The refiner particles should ideally be distributed uniformly in the melt, promoting as many nucleation events on the TiB_2_ particles as possible when solidification begins. As it can be observed, the presence of the non-equilibrium phases in inter-dendritic and around the grain structure due to the non-equilibrium solidification during casting led to the inhomogeneous chemical distribution and grain structure. It was previously established [38] that homogenization treatment could eliminate inhomogeneity of the chemical composition and microstructure inside the ingot. During homogenization, soluble intermetallic particles can dissolve into the matrix, forming a saturated grain with most elements. As a result, homogenization is an important process for ensuring alloys’ performance.

#### 3.1.3. DSC Analysis of the As-Casted Alloys

**Figure 4:**
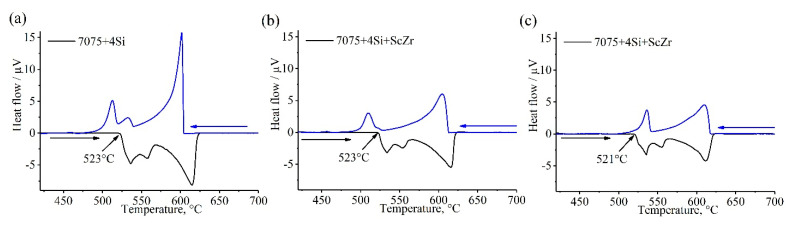
DSC curves of the investigated alloys; (**a**) 7075-4%Si, (**b**) 7075-4%Si + ScZr, and (**c**) 7075-4%Si + TiB.

DSC curves of as-cast alloys are shown in Figure 4. The melting temperature of the second phase during the homogenization annealing process can be measured through this analysis. The figure shows that the low eutectic phase will be dissolved initially when the temperature for homogenization is 523 °C in the 7075-4%Si and with Sc + Zr and 521 °C in the 7075-4%Si-Ti + B. So, the homogenization temperature must not exceed those temperatures. So, the selected temperature of homogenization annealing is 500 °C for 6.5 h.

**Figure 5:**
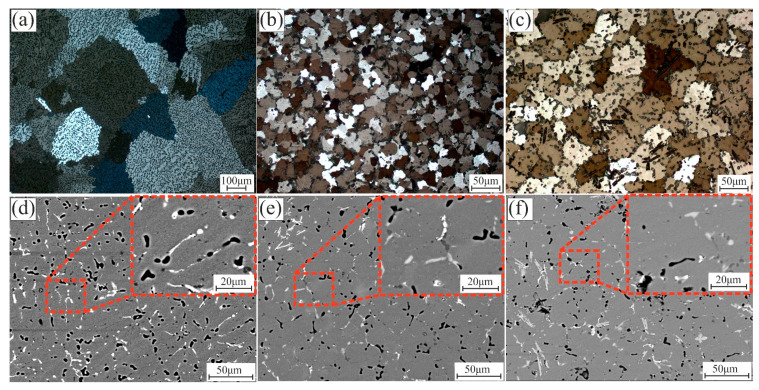
Microstructure of the homogenized alloys 500 °C for 6.5h: (**a**,**d**) 7075-4%Si, (**b**,**e**) 7075-4%Si + ScZr, and (**c**,**f**) 7075-4%Si + TiB.

According to the DSC analysis, the investigated alloys were homogenized at 500 °C for 6.5 h to dissolve the low melting temperature phases and perform stable alloys for the laser melting process. The SEM and OM of the modified alloys after homogenization annealing 6.5h are shown in Figure 5. In the figure, the grain boundaries became noticeably clearer and thinner where most of the residual phases dissolved and the dendritic structure was reduced. The distribution of the second phases around the grain boundaries became discontinuous. Moreover, there were few dendrites, and intermetallics phases still existed. The main reason for performing homogenization annealing pre-laser melting is to eliminate low-temperature phases around the grain boundaries, which causes cracks formation [39]. Furthermore, dissolving these phases into the grains to be fully saturated with all elements can enhance the LMZ characteristics and properties during laser melting.

### 3.2. Laser Melting Process

**Figure 6:**
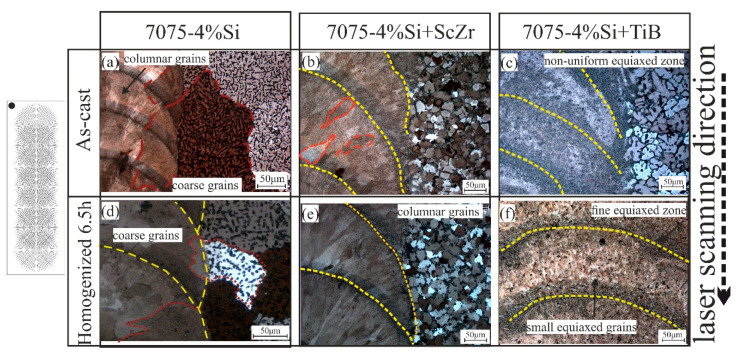
Optical micrographs (top view) of the LMZ for the as-casted and the homogenized samples; (**a**,**d**) 7075-4%Si, (**b**,**e**) 7075-4%Si + ScZr, and (**c**,**f**) 7075-4%Si + TiB.

After laser melting of the as-casted 7075-4%Si, the coarse grains near edges that crystallized from the melt pool and grew on a substrate of large grains of the base metal with a coarse columnar grains formation at the center of the track arranged in the direction of laser melting, as shown in Figure 6a. After laser melting the homogenized samples, a uniform structure was obtained due to dissolving most intermetallic phases into the matrix. The columnar grains were transformed into coarse grains at the center of the track (as shown in Figure 6d). In 7075-4%Si + ScZr, the modifying elements affected the structure, which the structure after laser fully transformed into columnar grains, but still, with different sizes. That’s because of the non-uniform distribution of elements in the as-casted samples; in the case of the homogenized samples, the structure became uniform columnar grains in the direction of laser (Figure 6b,e). In 7075-4%Si + TiB, the presence of Ti and B changed the nature of crystallization, and the columnar grain zones disappeared. All grains inside the LMZ were fine uniform and equiaxed grains due to the nucleation centers during solidification. The main difference between the laser-treated casted and homogenized samples is the uniformity and the distribution of the grains inside the LMZ (Figure 6c,f). And it can be noticed, Si has a significant impact on the absence of solidification cracks during laser melting. It can be concluded that the presence of Si reduces the sensitivity of the formation of hot cracks during solidification; and by adding the improved elements, whether Sc, Zr, Ti, and B, the nature of the alloy has been changed due to the formation of many nucleation centers inside the molten pool, which suppressed the formation of the columnar grains and the columnar grain transformed to equiaxed grain due to the rapid increase of the nucleation and growth rate of the equiaxed grain. This way eliminates its tendency of hot crack formation during rapid solidification.

**Figure 7:**
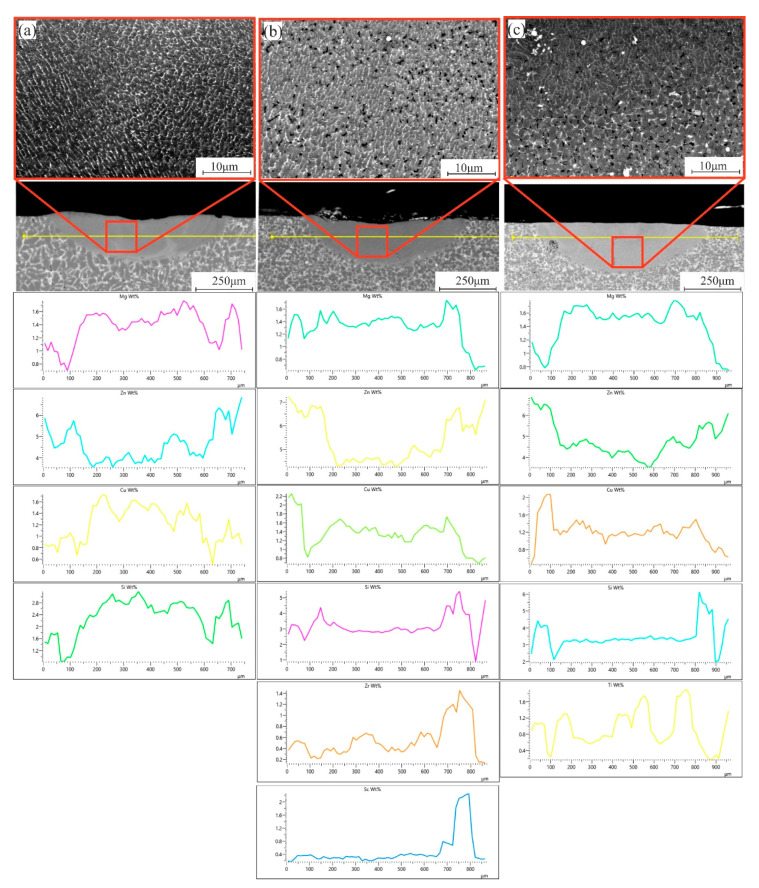
SEM micrographs and elemental distribution of the LMZ of the as-casted samples; (**a**) 7075-4%Si, (**b**) 7075-4%Si + ScZr, and (**c**) 7075-4%Si + TiB.

In the case of the as-casted samples, the SEM structure of the LMZ shows a free-defects structure but with nonuniformity in elemental distribution such Si, Cu, Zn, and Mg due to the melting structure with inhomogeneity distribution of elements and also evaporating Zn and Mg during the laser processing (Figure 7a) and this also affected the crystallization behavior as shown in the previous figure. The presence of the modifying elements positively affected the crystallization behavior, the amount of evaporation Zn and Mg, and the distribution of elements inside the melt pool due to the high amount of the nucleation centers such as Al_3_(Sc, Zr) and TiB_2_ (Figure 7b,c). However, the non-homogeneity of elements and intermetallic phases in the base alloy can affect the final properties and the new crystallized structure of the LMZ.

**Figure 8:**
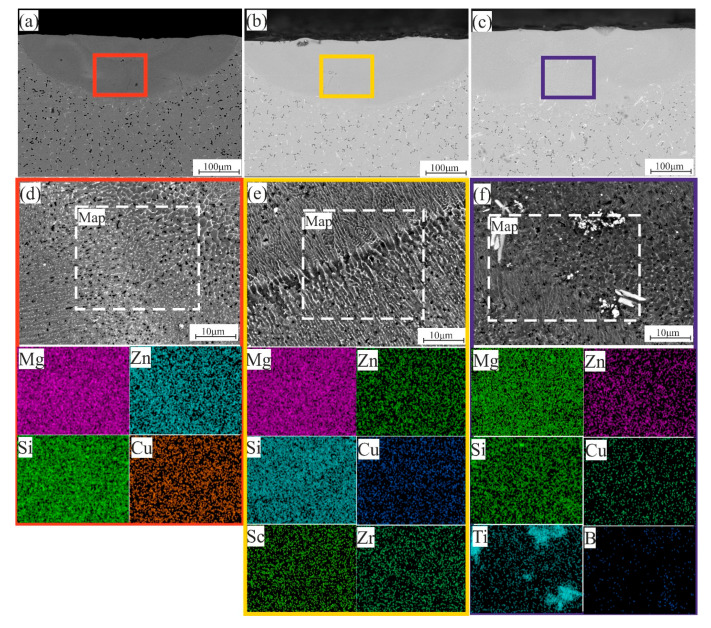
SEM micrographs and EDS maps analysis of the LMZ of the homogenized samples; (**a**,**d**) 7075-4%Si, (**b**,**e**) 7075-4%Si + ScZr, and (**c**,**f**) 7075-4%Si + TiB.

After the laser melting of the homogenized samples (Figure 8a–c), the nature of the structure became more uniform, as mentioned above due to dissolving the low-temperature phases and the saturated grains with the most elements. As shown in Figure 8d–f in the EDS map analysis, the LMZ has a fully uniform distribution of elements in all modified alloys and the presence of TiB_2_ particles in 7075-4%Si + TiB alloy, which enhanced the properties of the LMZ. So, the SEM and EDS analysis confirmed the idea that the laser treating of the homogenized samples performed more uniform and stable LMZ than the LMZ of the as-casted samples. This can be attributed to the LMZ of the homogenized samples having saturated grains with all elements.

### 3.3. Hardness Characteristics

**Figure 9:**
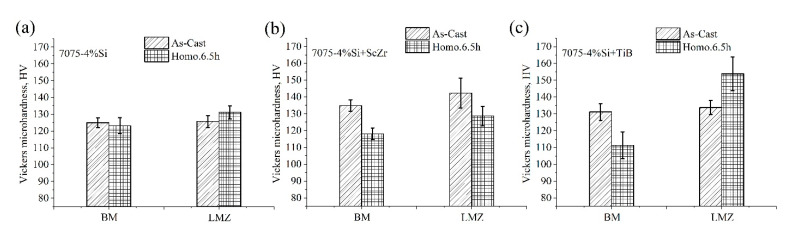
Vickers microhardness of the BM and the LMZ of the as-casted and homogenized alloys; (**a**) 7075-4%Si, (**b**) 7075-4%Si + ScZr, and (**c**) 7075-4%Si + TiB.

Hardness and microhardness values of the base metal and LMZ of the modified alloys in the as-cast and homogenized conditions were illustrated in Figure 9a–c. The hardness of the as-cast standard alloy with the addition of 4%Si was 125 ± 2.9 HV, and by adding 4%Si + TiB and 4%Si-ScZr, the hardness of the base metal increased to 134.9 ± 3.4 and 131.2 ± 5 HV, respectively. After homogenization of 500 °C for 6.5 h for all alloys, the hardness of the base metal was decreased to 123.2, 118.1, and 111.3 HV for 7075-4%Si, 7075-4%Si + ScZr, and 7075-4%Si + TiB, respectively, due to the dissolving of the low-temperature intermetallic phases during homogenization. After laser melting, the microhardness values of the LMZ of the as-casted and the homogenized samples were increased as compared to the base metal in all conditions. In the 7075-4%Si, the microhardness of the LMZ of the as-casted sample has an insignificant change, and the value is 125.7 ± 3.5 HV, but the LMZ of the homogenized samples was increased to 131.2 ± 3.9 HV. In 7075-4%Si + ScZr alloy, the LMZ increased compared to the base metal by 142.3 ± 5 and 128.7 ± 5.8 HV in the as-casted and homogenized conditions. After adding Ti and B to the 7075-4%Si, the LMZ increased to 133.8 ± 4.3 and 153.9 ± 5 HV in as-casted and homogenized conditions, respectively. The hardness values have positively increased due to Si addition and addition a combination of Ti + B and Sc + Zr. 

## 4. Conclusions

Aluminum alloy, AlZnMgCu (7075), was modified by adding 4%Si, 4%Si + ScZr, and 4%Si + TiB elements, then homogenized at 500 °C for 6.5 h. The effect of composition modification and homogenization annealing on the LMZ was studied. Microstructure analysis and microhardness evolution were investigated. From the results, we concluded that; 

In the as-cast state, the addition of 4%Si forms primary dendrites of the Al-rich solid solution with a coarse-grained dendritic structure with an average grain size of 297 ± 90 µm. After adding 4%Si-ScZr, the structure changed to a very fine nondendritic structure and decreased about 15.6 times according to the effect of the primary Al_3_(Sc, Zr) phase as a refining source and the suppression of grain growth. Similarly, adding 4%Si-Ti + B resulted in fine and uniform dendritic grains decreasing 10.2 times due to the soluble Al_3_Ti and TiB_2_, which act as potential nuclei for the primary phase nucleation.

After homogenization annealing, the grain boundaries became clearer and thinner where most of the residual phases dissolved, and the dendritic structure was reduced. The distribution of the second phases around the grain boundaries became discontinuous. After LMP of the as-casted samples, the 7075-4%Si exhibited coarse grains near edges that were crystallized from the melt pool. It grew on a substrate of large grains of the base metal with coarse columnar grains formation at the center of the track arranged in the direction of laser melting. In 7075-4%Si + ScZr, the structure after laser completely transformed into columnar grains, but in different sizes, because of the non-uniform distribution of elements of the as-casted samples. In 7075-4%Si + TiB, the presence of Ti and B changed the nature of crystallization, and the columnar grain zones disappeared.

After laser melting processing of the homogenized samples, homogenization 6.5 h of 7075-4%Si resulting in uniform structure and transform the columnar grains to coarse equiaxed grains in the center of the track, in the case of the homogenized 7075-4%Si + ScZr samples, the structure became uniform columnar grains in the direction of the laser. For 7075-4%Si + TiB, all grains inside the LMZ were small fine and equiaxed grains due to the nucleation centers during solidification. Generally, the hardness values of the BM and LMZ were increased by adding 4%Si, 4%Si + ScZr, and 4%Si-TiB compared to the same alloys without 4% Si and the modifiers. The LMP didn’t soften the LMZ of the as-casted samples; on the contrary, the hardness was slightly increased, and the hardness of the BM of the homogenized samples decreased. The LMZ exhibited higher hardness compared to the as-cast.

Finally, adding 4% Si with a combination of 1Ti + 0.2B and 0.29Sc + 0.4Zr to AlZnMgCu and homogenization annealing significantly enhanced the base alloy and the LMZ.

## Figures and Tables

**Table 1:** Chemical composition of the investigated alloys.

Alloy	Alloying Elements, wt.%										
	Zn	Mg	Cu	Cr	Mn	Si	Sc	Zr	Ti	B	Al
7075-4%Si	6.4	2.2	1.1	0.3	0.23	4					Bal.
7075-4%Si + ScZr	6.4	1.9	1	0.29	0.3	4.2	0.29	0.4			Bal.
7075-4%Si + TiB	6.7	1.8	1.2	0.26	0.3	4			1	0.2	Bal.

**Table 2:** Pulsed laser melting parameters.

Parameter	Unit	Value	Parameter	Unit	Value
power	V	300	Scanning speed	mm/s	1
Pulse Duration	ms	12	Overlap	mm	0.15
Shielding gas	Argon		Frequency	Hz	5
Pulse shape	Ramp-down		Laser diameter	mm	0.2–2.5

## Data Availability

The data presented in this study are available on request from the corresponding author. The data are not publicly available due to privacy.
